# The influence of nonrandom extra‐pair paternity on heritability estimates derived from wild pedigrees

**DOI:** 10.1111/evo.12649

**Published:** 2015-04-27

**Authors:** Josh A. Firth, Jarrod D. Hadfield, Anna W. Santure, Jon Slate, Ben C. Sheldon

**Affiliations:** ^1^ Department of ZoologyEdward Grey Institute, University of OxfordOxfordUnited Kingdom; ^2^ Institute of Evolutionary BiologyUniversity of EdinburghEdinburghUnited Kingdom; ^3^ Department of Animal and Plant SciencesUniversity of SheffieldSheffieldUnited Kingdom; ^4^ School of Biological SciencesUniversity of AucklandAucklandNew Zealand

**Keywords:** Extra‐pair copulations, misassigned paternity, pedigree error, pedigree simulation, quantitative genetics, SNP

## Abstract

Quantitative genetic analysis is often fundamental for understanding evolutionary processes in wild populations. Avian populations provide a model system due to the relative ease of inferring relatedness among individuals through observation. However, extra‐pair paternity (EPP) creates erroneous links within the social pedigree. Previous work has suggested this causes minor underestimation of heritability if paternal misassignment is random and hence not influenced by the trait being studied. Nevertheless, much literature suggests numerous traits are associated with EPP and the accuracy of heritability estimates for such traits remains unexplored. We show analytically how nonrandom pedigree errors can influence heritability estimates. Then, combining empirical data from a large great tit (*Parus major*) pedigree with simulations, we assess how heritability estimates derived from social pedigrees change depending on the mode of the relationship between EPP and the focal trait. We show that the magnitude of the underestimation is typically small (<15%). Hence, our analyses suggest that quantitative genetic inference from pedigrees derived from observations of social relationships is relatively robust; our approach also provides a widely applicable method for assessing the consequences of nonrandom EPP.

Estimating the heritability of traits in wild populations is fundamental in determining responses to selection, as well as understanding patterns of genetic variation (Lynch and Walsh 1998). Wild bird populations have been a popular subject for such work, as family structure and hence relatedness among individuals can often easily be inferred through observations during breeding attempts, and pedigrees built over multiple generations (Merilä and Sheldon 2001). However, molecular genetic techniques have often demonstrated the occurrence of extra‐pair paternity (EPP) in socially monogamous species (Griffith et al. 2002). Consequently, paternal links within the social pedigree may differ from those in the actual genetic pedigree, and therefore using social pedigrees for quantitative genetic parameter estimation may be problematic (Merilä et al. 1998; Keller et al. 2001; Charmantier and Reale 2005).

Initial investigations of the effect of pedigree error used empirical avian pedigrees, and compared estimates of heritability derived from midparent–offspring regressions using genetically correct pedigrees with those calculated from uncorrected pedigrees (Merilä et al. 1998; Keller et al. 2001). Although no substantial differences between social and genetic pedigree heritabilities were found, a trend of reduced heritabilities from social versus genetic pedigrees suggested that the error induced by EPP had the potential to decrease heritability estimates. Charmantier and Reale (2005) advanced this work by employing simulation techniques that bypassed potential underlying confounding effects within field data, and enabled consideration of how a range of EPP rates influence estimates of various heritabilities, which were derived using “animal models” (Henderson 1975). They showed that social pedigrees underestimate heritability most when EPP rates and trait heritabilities are high, as this increases the number of incorrect pedigree links, and causes extra‐pair young (EPY) to strongly resemble their genetic sire, therefore decreasing the average resemblance between putative paternal relatives (Charmantier and Reale 2005). Nevertheless, the extent of underestimation remained small and it was concluded that social pedigrees were generally reliable when EPP rates (≤20%) and trait heritabilities (0.1–0.4) were typical of those found in most bird species (Charmantier and Reale 2005).

Importantly, however, past work has assumed no relationship between the investigated trait and the misassigned paternities, and the extent to which this could decrease the accuracy of heritability estimates remains unknown (Keller et al. 2001; Charmantier and Reale 2005; Berenos et al. 2014). Indeed, a large body of research suggests that numerous morphological, behavioral, immunological, life‐history, and reproductive traits are associated with extra‐pair activity across many species of birds (Birkhead and Moller 1992; Moller and Ninni 1998; but see Akcay and Roughgarden 2007; Hsu et al. 2015). In such cases, this may result in additional bias in estimates of quantitative genetic parameters, as a result of the relationship between EPP and the trait being considered (Reid et al. 2014).

The primary objective of this study was to assess the validity of using social pedigrees for estimating the heritability of traits associated with EPP. Such traits may influence the gain, or loss (i.e., cuckoldry), of paternity in multiple ways. Therefore, first, we analytically derive how the trait values of the social and genetic fathers may influence heritability estimates from father–offspring regression. Second, we explore how animal model estimates of heritability are influenced by the degree, and mode, of nonrandom EPP within authentic populations using a permutation approach. As wild populations may differ in pedigree structure from both animal breeding designs and simple simulated pedigrees, which may also influence heritability estimation (Charmantier and Reale 2005; Quinn et al. 2006; Berenos et al. 2014), we utilized an extensive pedigree from a wild great tit (*Parus major*) population for which both a detailed social pedigree (gained through observations) and genetic pedigree (derived from large‐scale SNP genotyping) was available (and for which there has been extensive previous quantitative genetic work (McCleery et al. 2004; Garant et al. 2008; Liedvogel et al. 2012; Santure et al. 2013)). This population has a typical pedigree structure and rate of EPP (12.7–14%) similar to the average of most other socially monogamous bird species (∼11%; Blakey 1994; Griffith et al. 2002; Patrick et al. 2012). We combined this with simulation techniques that imposed five different EPP scenarios:

i Males with larger trait values gain more EPP (Hasselquist et al. 1996; Yezerinac and Weatherhead 1997; Johnsen et al. 1998).
ii Males with larger trait values suffer less cuckoldry (Smith et al. 1991; Kempenaers et al. 1992, 1997; Sheldon et al. 1997; Sheldon and Ellegren 1999).
iii Males with larger trait values gain more EPP and suffer less cuckoldry (Burley et al. 1996; Saino et al. 1997).
iv Males cuckold other males that are most dissimilar from themselves (Yasui 1998).
v Males cuckold other males that are most similar to themselves (Patrick et al. 2012; Forstmeier et al. 2014).

Through this, we highlight the extent to which social pedigrees (for which large quantities of data are available, and for which pedigree information is much easier, and frequently cheaper, to obtain) can be used in assessing heritability of traits that may correlate with paternal misassignment in wild pedigrees.

## Methods

### THEORY

Obtaining analytical results for the expected bias of animal model estimates would be challenging. However, analytical results for the expected bias of father–offspring regression estimates are possible. The difference between actual and estimated coefficients of kinship is at their most extreme for this type of comparison, and so these results should be considered as upper bounds on the degree of bias. The expected estimate of heritability from father–offspring regression is

(1) E[h2^]=2 cov z,o/ var z,

where *z* is the trait value of the social father and *o* the trait value of the offspring. Under the assumptions that (1) the social father's trait does not have a paternal effect; (2) there is no genetic correlation between the trait and female preference; and (3) there is no inbreeding, then  cov (z,o)= cov (z,a)/2, where *a* is the breeding value of the offspring's genetic father. If we assume that mating behavior is determined directly by the studied phenotype, then

(2) cov z,a=h2 cov z,z′,

where *z*' is the phenotype of the genetic father. Denoting EPP events as δ=1 (δ=0 otherwise ), where δ is also a random variable:

(3) cov z,a=h2 cov z,z1−δ+δe,

where *e* is the phenotype of the extra‐pair male (EPM), which is not defined when δ=0. It can be shown (see Supporting Information) that

(4) $$E\left[\hat{h^{2}}\right]=h^{2}\left[1+\bar{\delta }\gamma \left(\beta_{e|z}-1\right)+\;\beta_{\delta |z}\Delta \right],$$

where $\bar{\delta }$ is the mean EPP rate, γ is the ratio of the variance in phenotype of cuckolded males compared to all males, $\beta_{e|z}$ is the regression of EPM phenotype on social father phenotype, $\beta_{\delta |z}$ is the regression of being cuckolded on the social father phenotype, and Δ the average difference between the phenotypes of EPMs and the social fathers they cuckold. Perhaps surprisingly, equation (4) implies that heritability estimates under nonrandom EPP would not differ from those under random EPP if patterns and rates of EPP were only determined by the phenotypes of potential EPMs. Under these conditions, equation (4) simplifies to

(5) $$E\left[\hat{h^{2}}\right]=h^{2}-\bar{\delta }h^{2}$$

because there is no relationship between social father and EPM phenotypes ($\beta_{e|z}=0$), nor a relationship between social father phenotype and the probability of being cuckolded ($\beta_{\delta |z}=0$) and so necessarily γ=1. This result has been used previously to estimate $\bar{\delta }$ before the advent of cheap molecular markers (Alatalo et al. 1984). Under this scenario, animal model estimates should be biased downwards by no more than the EPP rate, as has been shown by previous simulation work (Charmantier and Reale 2005).

However, the bias is not simply determined by the EPP rate ($\bar{\delta }$) when patterns and rates of EPP are associated with the cuckolded male's phenotype. This can happen through two broad, but interacting, mechanisms. First, the phenotypes of EPMs and those they cuckold could be correlated, with a positive correlation $\left(\beta_{e|z}>0\right)$ reducing bias and a negative correlation ($\beta_{e|z}<0$) increasing bias. However, a lower bound on$\;\beta_{e|z}$ is −1 if the variances in trait value for all males, EPMs, and cuckolded males are approximately equal, and so heritability estimates from animal models are unlikely to be biased downwards by more than twice the EPP rate, and most likely substantially less.

A second mechanism is if the probability of being cuckolded depends on the social father's phenotype. Here, bias will increase when the probability of being cuckolded decreases with trait vale ($\beta_{\delta |z}<0$) and the phenotype of EPM is larger than the average (Δ>0) or vice versa. It should be noted that even if EPP is independent of EPM phenotype these two quantities will have different signs: if social fathers with low trait values get cuckolded ($\beta_{\delta |z}<0$) then by necessity EPMs (which are drawn randomly from the population) will have larger trait values (Δ>0). However, if EPP also depends on EPM phenotype then this is not necessarily the case: as an extreme example, imagine two types of male with phenotypes 0 and 1 and that males with phenotype 1 neither gain nor lose EPP, but males with phenotype 0 lose all their paternity (to each other). Under this example $\beta_{\delta |v}=-1$, yet Δ=0. Under these conditions $E\left[\hat{h^{2}}\right]=h^{2}$ (because γ=0) and there is no bias; this makes intuitive sense because the social father of every offspring has the same phenotype as the genetic father. In contrast, if males with phenotype 0 lost all their paternity to males with phenotype 1 then Δ=1 and $E[\hat{h^{2}}]=0$ generating large biases.

### STUDY SYSTEM

The empirical part of this study was conducted using the long‐term study population of resident great tits in Wytham Woods, Oxford, UK (51°46′N, 1°20′W; Perrins 1965). The area contains ∼1020 nestboxes in fixed positions which are visited regularly throughout the breeding season (April–May) to record breeding attempts and performance, and identify/capture adults (between days 6 and 14 of nestling phase) and nestlings (after day 15) to mark with a unique BTO (British Trust for Ornithology) metal leg ring. Blood has been collected for a limited subsample of adult birds between 1985 until 2004 and for a much larger proportion onwards. As the majority of studies attempting to estimate heritabilities usually focus on adult traits this pedigree, which consists only of adult birds (therefore giving the realized paternity for males), is appropriate for this work.

### PATERNITY ANALYSIS

A total of 2644 of the blood‐sampled individuals were chosen for genotyping on an Illumina iSelect BeadChip (SNP chip) of 9193 Single‐nucleotide polymorphism (SNP) (see Van Bers et al. 2012 for details). Following quality control, a linkage map of the great tit genome was constructed, with 4701 SNPs mapped to autosomes (van Oers et al. 2014). For computational reasons, a set of 1700 of the mapped SNPs were chosen for parentage analysis by selecting a third of evenly spaced SNPs on each chromosome to reduce interdependence. CERVUS 3.0 (Kalinowski et al. 2007) was employed to confirm social paternal pedigree links and identify previously unknown genetic links. For each offspring, the pool of candidate fathers included all genotyped males at least one year older than itself. Using 10,000 simulated mating events, paternity was assigned to the male with the highest paternity likelihood if they were assigned with high confidence (>99%) in CERVUS.

As this study was aimed at examining the possible effects of misassigned paternities, rather than data completeness (but see Charmantier and Reale 2005; Quinn et al. 2006) only individuals born between 2004 and 2009 (the period for which the large majority of data were available) and had both genetic parents known were included as offspring in the following analyses. Although this subset may have a reduced rate of EPP if social fathers are more likely to be sampled than EPMs, 12.5% of offspring were EPY. This is very similar to previous estimates based on analysis of paternity among nestlings within this population (Blakey 1994; Patrick et al. 2012). In total, the pedigree contained 1553 genotyped individuals, made up of 593 offspring and 960 founders (i.e., parents of these including genotyped males known to have sired young between 2004 and 2009).

### SIMULATIONS

We used simulation techniques to assess how the estimates of traits with heritabilities of 10, 30, 50, and 70% (a range spanning estimates often made for traits in wild populations) were influenced when linked to EPP through the five different mating scenarios. All analyses were run in R (R Development Core Team 2010). We generated traits of given heritabilities by simulating the phenotype for each individual down the genetic pedigree using standard methods (Lynch and Walsh 1998; Hadfield 2010). Then, to establish a given scenario of how the trait is linked to EPP (i.e., “EPP scenarios”—see Introduction), we used a permutation technique that maintained the structure of the observed genetic pedigree. This is desirable as pedigree structure may influence how paternity misassignment affects heritability estimates (Charmantier and Reale 2005; Quinn et al. 2006; Berenos et al. 2014), and this could potentially be exaggerated under nonrandom EPP. Thus, we simulated paternities conditional on the observed social pedigree and summary statistics regarding observed patterns of EPP (see below). We modeled the probability that the potential EPM *i* gains EPP in the brood of social father *j*, $E_{ij}$, following the logit‐linear model (Smouse et al. 1999; Hadfield et al. 2006):

(6) $$E_{ij}=\frac{e^{\lambda d_{ij}+\beta \left(z_{i,\;}z_{j}\right)}}{\sum_{m}\sum_{n\neq m}e^{\lambda d_{mn}+\beta \left(z_{m,\;}z_{n}\right)}},$$

where the denominator is the sum over all pairs of males (*m* and *n*) observed that year. To incorporate the likelihood that there are spatial constraints on the occurrence of EPP, $d_{ij}$ was defined as the distance between the potential EPM and the social father, and λ as the rate of decline of the probability of EPP with distance. We investigated five EPP scenarios with different functional forms for $f(z_{i},z_{j})$, where *z* is trait value:

- The trait of male *i*, that is, EPM trait values increase the probability of gaining EPP: $f\left(z_{i},z_{j}\right)=z_{i}$.
- The trait of male *j*, that is, social father trait values decrease the probability of being cuckolded: $f\left(z_{i},z_{j}\right)=-z_{j}$.
- A combination of (i) and (ii): $f\left(z_{i},z_{j}\right)=\;z_{i}-\;z_{j}$.
- The absolute difference in trait values between the EPM and social father increases the probability of EPY within this brood: $f\left(z_{i},z_{j}\right)=\;\left|z_{i}-\;z_{j}\right|$.
- The absolute difference in trait values between the EPM and social father decreases the probability of EPY within this brood: $f\left(z_{i},z_{j}\right)=\;-\left|z_{i}-\;z_{j}\right|$.

Finally, β represents the strength of the effect. Smouse et al. (1999) state in passing that β is analogous to the selection gradients defined by Lande and Arnold (1983). We show that this can be justified under our scenarios [i]–[iii] when the number of candidate fathers is large (Supporting Information).

We simulated the trait to have a standard deviation (SD) of one so that β can be roughly interpreted as a standardized selection gradient (note that β must be multiplied by two in scenario [iii] to get the standardized selection gradient).

Under each scenario, β was set as either 0, 0.1, 0.2, 0.4, 0.8, or 1.6; these values range from no association between the trait and EPP (or “zero selection”) to strengths of association (or “selection”) well above the range typically encountered in wild populations.

A number of extra‐pair (EP) events (equal to the number observed in the genetic pedigree) were then drawn from the distribution defined by equation (6). However, note that this distribution is not multinomial because a social father can only be subject to one EP event (i.e., social fathers are sampled without replacement). Whether offspring are sired by an EPM is nonindependent within broods (Brommer et al. 2007; Morrissey and Wilson 2010). Therefore, at each successful EP event, one offspring was randomly assigned to the EPM. Then, the remaining offspring were assigned to the EPM given the known probability of additional young within an EP event were also EPY. Upon assigning individuals as EPY, their trait value was again calculated using the standard methods (Lynch and Walsh 1998; Hadfield 2010). Each combination of heritability, EPP scenario, and strength of the scenario (i.e., strength of relationship between the trait and EPP) within this was simulated 100 times, generating new starting traits on each occasion. We also calculated the statistics γ, βe|z,βδ|z, and Δ identified as important in the analytical model (eq. 4). Further, to allow comparison to previous studies of EPP, we also report the difference (in terms of the number of SDs) between the traits of all EPMs and those of cuckold males. As the differences between these groups of males may influence the total SD, we use the SD of the group with the largest sample size.

### HERITABILITY ESTIMATES

For the social, observed genetic, and simulated genetic pedigrees, the additive genetic variance ($V_{A}$) and residual variance ($V_{R}$) (Falconer and Mackay 1996) were estimated using the animal model (Henderson 1975; Lynch and Walsh 1998; Kruuk 2004) following a Bayesian Markov chain Monte Carlo method (Hadfield 2010). To enable the large number of simulations to be carried out, chains were run for 23,000 iterations after a burn‐in of 3000 with a thinning interval of 10. The prior distribution for $V_{A}$ was as a scaled 1000 *F*_1,1_‐distribution, and a flat improper prior was placed on $V_{R}$. Following this, narrow sense heritability estimates were calculated using the standard formula (Falconer and Mackay 1996). For each heritability (*n* = 4), EPP scenarios (*n* = 5), and different strength of these scenarios (*n* = 6), 100 simulated pedigrees were generated and analyzed.

## Results

### SIMULATED PEDIGREES

**Figure 1:**
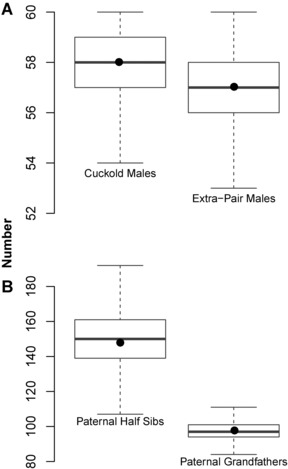
Measures of pedigree structure related to paternity in (A) number of individuals and (B) number of links, from permuted pedigrees (box plots) and observed genetic pedigree (dots).

As we incorporated multiple parameters influencing the patterns of EPP observed in the genetic pedigree into our permutations of the social pedigree (see Methods), our genetic pedigrees simulated with no association between the male trait and EPP (i.e., zero selection) did not differ from the observed genetic pedigree in measurements of pedigree structure that could be influenced by such techniques (Fig. 1). This ensured that the simulation technique only applied differences through imposing a nonrandom relationship between the male trait and EPP. By not independently changing the underlying pedigree structure, we avoided the implications that this could have on heritability estimates.

**Figure 2:**
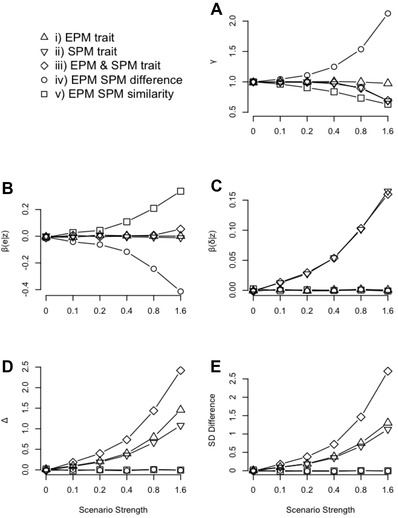
The mean value of the pedigree statistics shown to be important to the analytical model (eq. 4). The *x*‐axis shows the strength of the scenario (i.e., strength of relationship between the trait and EPP) which refers to β from equation (6). This is analogous to a linear standardized selection gradient for scenarios [i], [ii], and [iii] (Supporting Information). Trait heritability had no influence on these parameters so simulations of the same scenario and selection strength are pooled for clarity. (A) γ is the variance in phenotype of cuckolded males compared to all males, (B) β_(_ *_e_*_|_ *_z_*_)_ is the regression of EP male phenotype on social male phenotype, (C) β_(δ|_ *_z_*_)_ is the regression of being cuckolded on the social male phenotype, (D) Δ is the average difference between the phenotypes of EP males and the social males they cuckold. Finally, (E) shows the average difference between the phenotypes of all EP males and of all cuckold males (in terms of number of standard deviations ‐ where standard deviation is calculated from the group with the largest sample size). However, the statistics in (E) are not directly included in the analytical model (eq. 4).

The statistics identified as important in the analytical model (eq. 4) showed the expected trends given the different scenarios (Fig. 2). For example, when males lose paternity to others most dissimilar from themselves (scenario [iv]), the variation in cuckolded males phenotypes (γ) increases (as males with the most extreme traits are likely to lose paternity; Fig. 2A). Further, the relationship between the social fathers trait and the male that cuckolds him ($\beta_{e|z})$ becomes more negative in this scenario, yet, when males cuckold others most similar from themselves (scenario [v]), $\beta_{e|z}$ increases (Fig. 2B). Similarly, a relationship between the male's trait and suffering cuckoldry ($\beta_{\delta |z}$) is only generated when this was directly simulated (scenarios [ii] and [iii]; Fig. 2C). Finally, the mean difference between an EPM and the male he cuckolded (Δ) increased when the trait was either positively related to gaining EPP (scenario [i]) or negatively related to suffering cuckoldry (scenario [ii]), but was largest when both of these processes were in play simultaneously (scenario [iii]) (Fig. 2D).

### HERITABILITY ESTIMATES

**Figure 3:**
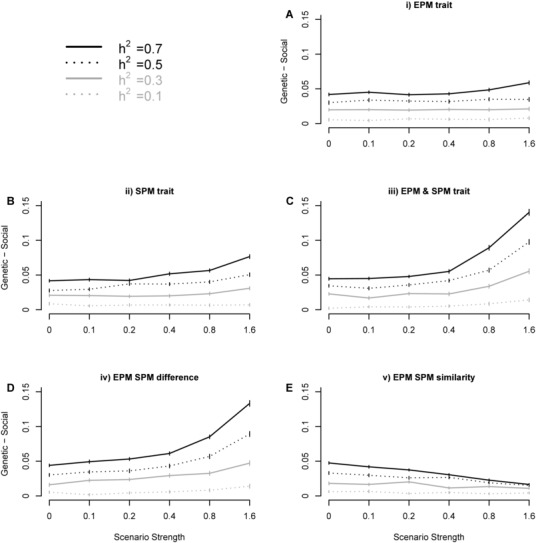
Total difference between the heritability estimates using the genetic pedigree in comparison to the social pedigree. Titles illustrate EPP scenario: (A) EPM trait increases EPP, (B) social father trait decreases cuckoldry, (C) combination of i and ii, (D) difference between EPM and social father increases cuckoldry, and (E) difference between EPM and social father decreases cuckoldry, (see Methods for details). Vertical lines indicate standard error whereas mid‐point represents the mean. Difference is expressed as difference from input heritability and proportion difference (%) is shown in Figs. S1 and S2, respectively. The *x*‐axis shows the strength of the scenario (i.e., strength of relationship between the trait and EPP) which refers to β from equation (6). This is analogous to a linear standardized selection gradient for scenarios [i], [ii], and [iii] (Supporting Information).

Heritability estimates generated using social pedigrees were generally lower than those accurately derived from genetic pedigrees (Figs. 3, S1). This effect is most pronounced at high levels of heritability, although when expressed as a percentage difference, this pattern is not observed (as the analytical results suggest) (Fig. S2). Heritability underestimation does not increase with increasing selection for males with larger traits to gain EPP (scenario [i]; Fig. 3A). The slight increase observed for traits with high heritability is an artifact of simulating strong selection, as in these extreme circumstances, inhibiting a male from gaining EPP at his own brood causes males with low trait values to be more likely to be cuckolded (eq. 6). Indeed, heritability underestimation is increased when low trait values are related to cuckoldry but this effect is only observed at high strengths of selection and heritability >10% (Fig. 3B). When these two scenarios act in combination, heritability is underestimated even further (Fig. 3C) as the EPMs differ more from those they cuckold (Fig. 2D). This can result in >10% raw heritability drop (or 20% proportional decrease) at the most extreme points, which is similar to scenario [iv] (i.e., males cuckold others most dissimilar from themselves; Figs. 3D, S2). However, the increased underestimation in scenario [iv] is driven primarily by the negative relationship between the EPMs trait and the trait the male they cuckold (Fig. 2B). Notably, this relationship becomes positive if males cuckold others similar to themselves (scenario [v]), causing heritability underestimation to decrease beyond what is expected under random EPP (Fig. 3E).

## Discussion

By combining an analytical approach with simulations using empirical data, we investigated how a nonrandom association between a trait and paternity misassignment may influence heritability estimates over a range of potential scenarios. We confirm that EPP generally causes social pedigrees to slightly underestimate heritability (Keller et al. 2001; Charmantier and Reale 2005). Crucially, however, we demonstrate that this can be influenced by the relationship between the focal trait and the occurrence of EPP (Fig. 3), and we highlight the mechanisms underlying this (eq. 4; Fig. 2).

Much literature links particular male characteristics to the gain of EPP (Griffith et al. 2002), but, interestingly this is not predicted to decrease heritability estimates (gained from parent–offspring regression) more than expected under random EPP (eq. 4). This is supported from simulations using animal models to estimate heritability (Figs. 2, 3). It is also commonly found that particular traits may relate to the likelihood of cuckoldry, for example, in our study species (great tit) for longevity, physical, and behavioral traits (Blakey 1994; Lubjuhn et al. 2007; Kawano et al. 2009; Patrick et al. 2012). Analytically, it is expected that this relationship may increase underestimation of heritability from social pedigrees (eq. 4; Fig. 2B), yet the empirically parameterized simulations illustrate that the effect is minor even when the selection acting through EPP is 0.8 or above. Although selection gradients are rarely calculated and reported in literature surrounding EPP, such high values are generally likely to be uncommon (Kingsolver et al. 2001). This selection was found to be equivalent to ∼0.6 SD difference between the trait values of cuckolded males and those gaining EPP (Fig. 2E), which is much higher than found for great tit breast stripe (0.16 SDs difference—Kawano et al. 2009), yet lower than some more extreme examples in related species, such as timing of blue tit (*Cyanistes caeruleus*) song (0.8 SDs difference—Poesel et al. 2006).

These first two EPP scenarios may act in combination, and this has been reported in multiple populations, although its prevalence is still debated (Akcay and Roughgarden 2007; Hsu et al. 2015). Under this scenario, heritability was underestimated by ∼10% (Fig. 3; proportional to a ∼13% total reduction—Fig. S2) when selection was 0.8 or above, therefore differing rather substantially from just a ∼5% (proportionately 7%) underestimate under random EPP (Figs. 3; S1). Similarly, this was also observed in a scenario where individuals cuckold others that are most different from themselves. This could, for instance, be generated by female efforts to increase within‐brood diversity (Yasui 1998) or the existence of competing behavioral strategies that are most vulnerable to cuckoldry by one another. As this scenario requires no overall difference in trait values between the males that gain EPP and those that are cuckold (Fig. 2D, E), it may be difficult to detect, despite its ability to underestimate heritability by almost 20% (proportionally—Fig. S2). Such underestimation is significantly larger than compared with the findings of Charmantier and Reale (2005), who concluded traits with heritability of 0.4 in large pedigrees with 10% EPP rates are underestimated by 0.8–6.6%.

Finally, males could be cuckolded by those most similar to themselves if, for instance, EPP was nonadaptive (Forstmeier et al. 2014) and females simply maintained their mating preferences over both social and genetic mate choices. This scenario could also arise if particular phenotypes were associated with both the loss, and gain, of paternity through EPP. For example, in our study system, great tits with “bold” personalities suffered increased cuckoldry but gained more EPP (Patrick et al. 2012). Encouragingly, this scenario decreases the underestimation of heritability by the social pedigree, as the genetic father is similar to the social father. Therefore, here, the social pedigree can be used more reliably than expected under random EPP. It is also notable that the underestimation of heritability for traits with low (10%) heritability does not appear to increase under any scenario (Fig. 3). Thus, traits with low heritability (e.g., fitness components) appear unlikely to be greatly underestimated through using social pedigrees.

The error rate resulting from EPP in the great tit social pedigree used in this study (12.5%) is very close to the average EPP rate of socially monogamous bird species (∼11%, Griffith et al. 2002); therefore these findings may be reasonably applicable to a large number of systems. Although the analytical model provided (eq. 4) allows consideration of a large range of EPP rates, the empirically parameterized simulation approach does not. Therefore, applying the same methodology to other pedigrees would now be beneficial to confirm that the general patterns are also consistent under more extreme rates of EPP. This may be facilitated through open‐source pedigree permutation software, such as *Pedantics* (Morrissey et al. 2007; Morrissey and Wilson 2010), as well as the R code provided here (Supporting Information). Furthermore, although random pedigree errors may have similar effects on genetic variances and covariances, they may have a larger influence on estimates of parental and indirect genetic effects (Morrissey et al. 2007). Indeed, the influence of nonrandom pedigree error on other quantitative genetic parameters remains largely unknown, yet it has the potential to influence estimates of inbreeding (Reid et al. 2014), genetic correlations and covariances (Berenos et al. 2014), and indirect effects, for example, if males alter their behavior in different ways in response to EPP (Eliassen and Kokko 2008). The analytical model (eq. 4) could potentially be expanded to incorporate these other quantitative genetic parameters. Similarly, particular caution should also be taken when utilizing multiple measures that could be subject to pedigree error (Reid et al. 2014), as in the case of estimating the response to selection of a trait that is associated with EPP, where both heritability and selection could be underestimated. Finally, although the biases we report are small, it is possible that comparative/meta‐analytic studies of heritability may systematically bias certain comparisons. For example, when testing differences in heritability between sexually and nonsexually selected traits (Alatalo et al. 1997), the possibility that the effects are driven by biases in estimation should be acknowledged.

In conclusion, although pedigree errors in wild populations only result in minor underestimation of heritability of particular traits (Keller et al. 2001; Charmantier and Reale 2005; Berenos et al. 2014), an association with EPP may influence this. However, traits of low heritability appear to be relatively unaffected by this, and, even for traits with higher heritability, the social pedigree remains adequate in all but the most extreme scenarios. This demonstrates the general utility of social pedigrees under most circumstances, although further consideration of multiple systems and various quantitative genetic parameters is now needed to guide our level of assurance in utilizing long‐term social pedigrees in understanding evolutionary processes.

## Supporting information

Supplementary Information
