# Supplementary material for: The influence of nonrandom extra‐pair paternity on heritability estimates derived from wild pedigrees
Source: Evolution. 2015 Apr 27;69(5):1336–44. doi: 10.1111/evo.12649 (PMC4950017; doi:10.1111/evo.12649)
Supplement: Supplementary file 1 — Supplementary Information [file EVO-69-1336-s001.docx]

**Supplementary Information**

*Derivation of Equation 4 in Main text:*

$$COV\left( z,a \right)= h^{2}COV\left( z,z\left( 1-\delta\right)+\delta e \right)$$

where z, e, and $\delta$ are random variables. From the law of total covariance:

$COV\left( z,\delta e \right)= E_{\delta}\left[ COV\left( z,\delta e | \delta\right) \right]+COV_{\delta}(E\left[ z|\delta\right], E[\delta e|\delta])$

where the outer expectations and covariances are subscripted with $\delta$ to make it clear what is being averaged over. Given $\delta e$ is a constant when $\delta=0$, then

$$COV\left( z,\delta e \right)= \bar{\delta}COV\left( z,e | \delta=1 \right)+COV_{\delta}\left( E\left[ z|\delta\right], E\left[ \delta e | \delta\right] \right)= \bar{\delta}COV\left( z,e | \delta=1 \right)+E\left[ z | \delta=1 \right]\bar{\delta}E\left[ e | \delta=1 \right]-E\left[ z \right]\bar{\delta}E\left[ e | \delta=1 \right]= \bar{\delta}COV\left( z,e | \delta=1 \right)+\left( E\left[ z | \delta=1 \right]-E\left[ z \right] \right)\bar{\delta}E\left[ e | \delta=1 \right]= \bar{\delta}COV\left( z,e | \delta=1 \right)$$

$$+\bar{\delta}(1-\bar{\delta})(E\left[ z | \delta=1 \right]-E\left[ z | \delta=0 \right])E[e|\delta=1]$$

Noting $E\left[ z | \delta=1 \right]-E\left[ z | \delta=0 \right]$ is the regression of z on $\delta$ and $\bar{\delta}\left( 1-\bar{\delta} \right)$ is the variance of $\delta$, then:

$$COV\left( z,\delta e \right)= \bar{\delta}COV\left( z,e | \delta=1 \right)+COV(z,\delta) E[e|\delta=1]$$

Following the same logic:

$$COV\left( z,\delta z \right)=\bar{\delta}COV\left( z,z | \delta=1 \right)+COV(z,\delta) E[z|\delta=1]$$

to give

$$COV\left( z,z\left( 1-\delta\right)+\delta e \right)= VAR\left( z \right)-\bar{\delta}COV\left( z,z | \delta=1 \right)+COV\left( z,\delta\right)\left[ E\left[ e | \delta=1 \right]-E\left[ z | \delta=1 \right] \right]+ \bar{\delta}COV\left( z,e | \delta=1 \right)=VAR\left( z \right)-\bar{\delta}COV\left( z,z | \delta=1 \right)+COV\left( z,\delta\right)\Delta+ \bar{\delta}COV\left( z,e | \delta=1 \right)$$

and so

$$\hat{h^{2}}=\frac{COV\left( z,a \right)}{VAR\left( z \right)}=h^{2}[1-\bar{\delta}\frac{COV\left( z,z | \delta=1 \right)+COV\left( z,e | \delta=1 \right)}{VAR\left( z \right)}+\beta_{\delta|z}\Delta]$$

multiplying the numerator and denominator of the fraction by $\mathrm{VAR}\left( z | \delta=1 \right)=COV\left( z,z | \delta=1 \right)$ gives:

$$\hat{h^{2}}=h^{2}[1-\bar{\delta}\gamma(1+\beta_{e|z})+\beta_{\delta|z}\Delta]$$

where $\gamma$ is the ratio of the variance in cuckolded male phenotypes compared to all males.

*Supplementary Information regarding Equation 6 in Main Text*

$\beta$ in Equation 6 represents the strength of the effect. Smouse et al (1999) state in passing that $\beta$ is analogous to the selection gradients defined by Lande & Arnold (1983). Under our scenarios i) - iii), this can be justified when the number of candidate fathers is large. The selection gradient as defined in Lande & Arnold (1983) is:

$\beta=\frac{\int p\left( z \right)W'(z)dz}{\int p(z)W(z)dz}$

When W(z) has the form $\exp\left( z\beta+\eta\right)/\tau$ where $\eta$ and $\tau$ do not depend on z, $\beta W(z)= W'(z)$. This is the form of a GLM with log-link, rather than the multinomial GLM with logit-link defined in Equation 6. However, as the number of males contributing to the denominator in Equation 6 increases, the Equation tends to the form $\exp\left( z\beta+\eta\right)/\tau$ as the contribution of $\exp\left( z\beta+\eta\right)$ to $\tau$ diminishes.


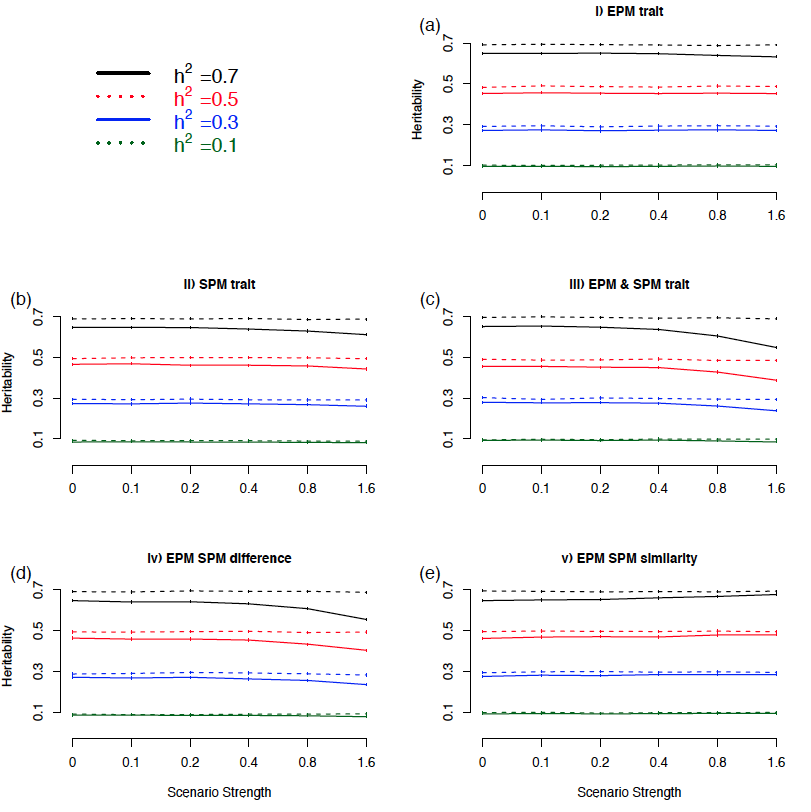


**Figure S1** Estimates of heritability using the social pedigree (unbroken lines) and genetic pedigree (dashed lines). Titles illustrate EPP scenario : a) EPM trait increases EPP, b) SPM trait decreases cuckoldry, c) Combination of i & ii, d) Difference between EPM and SPM increases cuckoldry and e) Similarity between EPM and SPM increases cuckoldry (see Methods for details). Traits generated at 0.7, 0.5, 0.3 and 0.1 heritability are shown in black, red, blue and green respectively. Vertical lines indicate standard error whilst mid-point represents the mean. The strength of the scenario (i.e. strength of relationship between the trait and EPP) is shown on x-axis.

**
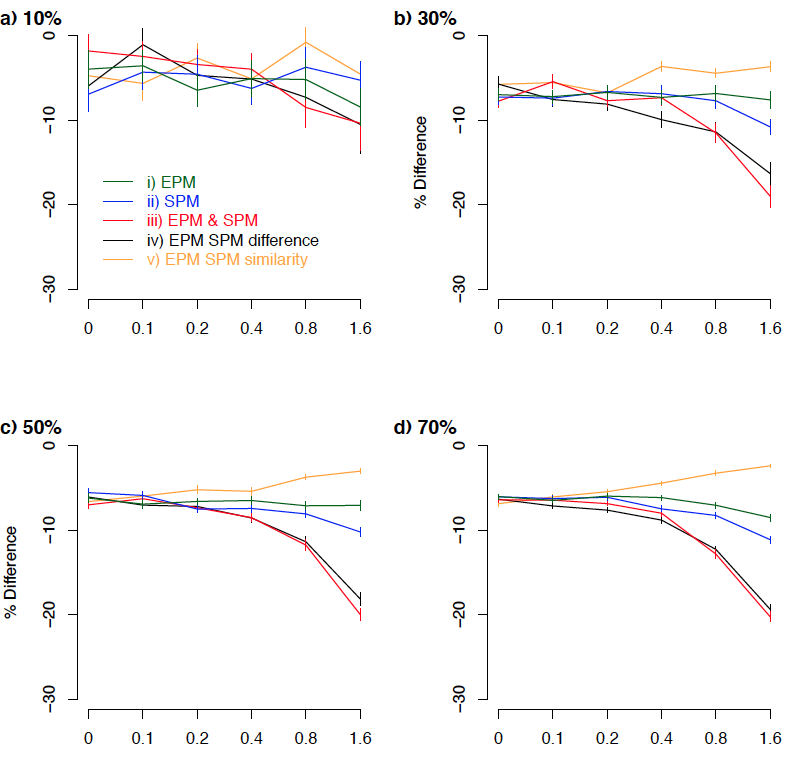
**

**Figure S2** Percentage difference between heritability estimates of social pedigree in comparison to when the genetic pedigree was used. Titles indicate the simulated trait heritability (a) 0.1, (b) 0.3, (c) 0.5, and (d) 0.7. Line colour shows scenario of selection green = EPM trait increases EPP, blue = SPM trait decreases cuckoldry, red = Combination of i & ii, black = Difference between EPM and SPM increases cuckoldry, orange = Similarity between EPM and SPM increases cuckoldry (see Methods for details). Vertical lines indicate standard error whilst mid-point represents the mean. The strength of the scenario (i.e. strength of relationship between the trait and EPP) is shown on x-axis.

*R Code for running Simulations – associated data available through Dryad*

*#Non-random EPP pedigree simulation*

*library(MCMCglmm);library(pedantics)#load library*

*#Load in the pedigree*

*a<-load(file="ped_and_males.RData") #loads 2 objects: 'ped' is the full pedigree, 'males' contains all the males that could have sired epy each year*

*#write two functions that are needed in the loop:*

*dist2<-function(from.this,to.these){(as.matrix(dist(rbind(from.this,to.these)))[-1,1])} # getting the distance from 1 point to multiple other points*

*sample.ep.events<-function(male.traits){*

*pmat<-exp((male.traits*selection)+(dist.m*EPPlambda))*

*#for the cells for a social father gaining epp at his own brood, set this to zero:*

*pmat[cbind(1:nrow(pmat),match(broods$dad,colnames(pmat)))]<-0*

*pmat<-pmat/sum(pmat) #scale probabilities*

*#sample some broods to contain epp:*

*epbroods<-sample(broods$brood,nbroods,prob=rowSums(pmat))*

*#determine which males sired young that the ep broods*

*rownames(pmat)<-broods$brood*

*apply(pmat[as.character(epbroods),],1,function(a)sample(colnames(pmat),1,prob=a))*

*} #function returning the sampled ep events given the particular scenario (as the influence of the male trait on epp changes with the different scenarios)*

*#Set values for simulation:*

*agv<-0.1 #set the additive genetic variance*

*ag.m<-0 #set mean for trait*

*rv<-1-agv #set residual variance*

*r.m<-0 #mean of residual variance*

*selection<-0.1 #for selection strength*

*scenario<-"abs(EPM-SPM)"#The following choices of sexual selection scenario:*

*#"EPM" = increased EPM traits = increased chance of gaining EPP*

*#"SPM" = decreased SPM traits = increased chance of cuckoldry*

*#"EPM-SPM" = a combination of the above*

*#"abs(EPM-SPM)" = males cuckold others who have trait values most dissimilar to themselves*

*#"-abs(EPM-SPM)" = males cuckold others who have trait values most similar to themselves*

*propEPC<-0.423 #given that one chick in a brood is EPY, the proportion of the other chicks that are EPY*

*EPPlambda<--0.0129 #relationship between distance and EPP*

*#make a dataframe to store breeding values in, generated down the true genetic pedigree, assigning all founders with random traits*

*all.phen<-data.frame(id=ped$id)*

*all.phen$bv<-rbv(orderPed(ped[,c("id","dam","dad")]),G=agv) #generates bv of trait down the pedigree - here, we use the social pedigree, as new breeding values are assigned depending on how the pedigree is permuted*

*all.phen$r<-rnorm(nrow(all.phen),r.m,sqrt(rv)) #generates r*

*all.phen$phen<-all.phen$r+all.phen$bv #all phenotypic values*

*years<-sort(unique(ped$year))*

*#carry out a permutation for each year:*

*for (i in years){*

*#find the broods of this year:*

*broods<-unique(ped[ped$year==i & !is.na(ped$year),c("brood","dad","lat","long")])*

*#assign social male phenotype to brood:*

*broods$phen<-all.phen$phen[match(broods$dad,all.phen$id)]*

*#find the males that where breeding:*

*epmales<-males[males$year==i,]*

*#add their phenotype:*

*epmales$phen<-all.phen$phen[match(epmales$id,all.phen$id)]*

*#now, for use in the log-linear model to choose EP events, create 3 matrices of broods (rows) by ep males (columns).*

*#i) the ep males trait value (all cells in a column are identical)*

*epm.t<-matrix(epmales$phen,nrow(broods),nrow(epmales),byrow=T,dimnames=list(broods$id,epmales$id))*

*#ii) the social males trait value (all cells in a row are identical)*

*spm.t<-matrix(broods$phen,nrow(broods),nrow(epmales),dimnames=list(broods$id,epmales$id))*

*spm.t<-(spm.t)*-1 #'*-1' so that larger values are associated with decreased cuckoldry risk*

*#iii) the distance between brood and potential epm*

*dist.m<-t(apply(broods[,c("lat","long")],1,dist2,epmales[,c("lat","long")]))*

*#now we have these 3 matrices, we can calculate the probability of every possible ep event (i.e. male gaining paternity at a brood) for the scenario under consideration and sample this given the no. of ep broods that year*

*nbroods<-length(unique(ped$brood[ped$brood %in% broods$brood & ped$dad!=ped$sire]))#no. of ep broods that year*

*#scenario specific part (determine which ep events occur, given which scenario is under consideration):*

*if(scenario=="EPM")epevents<-sample.ep.events(epm.t) #the trait of the EPM and the distance from the brood governs the probability of EPP*

*if(scenario=="SPM")epevents<-sample.ep.events(spm.t) #the trait of the SPM and the distance of the EPM from the brood governs the probability of EPP*

*if(scenario=="EPM-SPM")epevents<-sample.ep.events(epm.t+spm.t) #the trait of the SPM and both the trait of the EPM and his distance from the brood governs the probability of EPP*

*if(scenario=="abs(EPM-SPM)")epevents<-sample.ep.events(abs(epm.t+spm.t))*

*#the difference between the trait of the SPM and the trait of the EPM, along with the distance of the EPM from the brood, governs the probability of EPP*

*if(scenario=="-abs(EPM-SPM)")epevents<-sample.ep.events((-abs(epm.t+spm.t)))*

*#the similarity between the trait of the SPM and the trait of the EPM, along with the distance of the EPM from the brood, governs the probability of EPP*

*#Now EP events have been chosen(i.e. which broods are cuckold by which males), we choose which chicks are EPY (at least 1 chick is EP, and the remaining chicks are assigned as EPP given the probability of a sibling of an EP chick also been sired by the EP father)*

*all.young<-ped[ped$brood%in%names(epevents),] #finds all chicks from the chosen ep broods*

*epchicks<-unlist(tapply(all.young$id,all.young$brood,function(a)a[as.logical(rbinom(length(a),1,prob=sample(c(1,rep(propEPC,length(a)-1)))))])) #for each brood, randomly samples 1 chick with probability 1, and the remaining with probability equal to 'propEPC' - so now we have which chicks were ep*

*#make the changes to the pedigree*

*#i) revert all offspring that year to be sired by their social father*

*ped$sire[ped$year==i & !is.na(ped$year)]<-ped$dad[ped$year==i & !is.na(ped$year)]*

*#ii) assign the selected ep chicks to their ep sire:*

*ped$sire[ped$id %in% epchicks]<-epevents[as.character(all.young$brood[match(epchicks,all.young$id)])]*

*#iii) assign the new breeding values and phenotype to these:*

*mumbv<-all.phen$bv[match(ped$dam[ped$id %in% epchicks],all.phen$id)]*

*dadbv<-all.phen$bv[match(ped$sire[ped$id %in% epchicks],all.phen$id)]*

*iver<-inverseA(orderPed(ped[,c("id","dam","sire")]))*

*dii<-iver$dii[match(ped$id,iver$pedigree$id)]*

*dii<-dii[ped$id %in% epchicks]*

*all.phen$bv[match(ped$id[ped$id %in% epchicks],all.phen$id)]<-rnorm(length(mumbv),(mumbv+dadbv)/2,sd=sqrt(agv*dii))*

*all.phen$phen<-all.phen$bv+all.phen$r*

*} #ends year loop*
